# Audience effects in the Atlantic molly (*Poecilia mexicana*)–prudent male mate choice in response to perceived sperm competition risk?

**DOI:** 10.1186/1742-9994-6-17

**Published:** 2009-08-21

**Authors:** Madlen Ziege, Kristin Mahlow, Carmen Hennige-Schulz, Claudia Kronmarck, Ralph Tiedemann, Bruno Streit, Martin Plath

**Affiliations:** 1 Department of Ecology & Evolution, J.W. Goethe University Frankfurt, Siesmayerstrasse 70-72, D-60054 Frankfurt am Main, Germany; 2 Institute of Biochemistry & Biology, Unit of Evolutionary Biology & Systematic Zoology, University of Potsdam, Karl-Liebknecht Str. 24-25, 14476 Potsdam, Germany

## Abstract

**Background:**

Multidirectional interactions in social networks can have a profound effect on mate choice behavior; e.g., *Poecilia mexicana *males show weaker expression of mating preferences when being observed by a rival. This may be an adaptation to reduce sperm competition risk, which arises because commonly preferred female phenotypes will receive attention also from surrounding males, and/or because other males can copy the focal male's mate choice. Do *P. mexicana *males indeed respond to perceived sperm competition risk? We gave males a choice between two females and repeated the tests under one of the following conditions: (1) an empty transparent cylinder was presented (control); (2) another ("audience") male inside the cylinder observed the focal male throughout the 2^nd ^part, or (3) the audience male was presented only before the tests, but could not eavesdrop during the actual choice tests (non-specific sperm competition risk treatments); (4) the focal male could see a rival male interact sexually with the previously preferred, or (5) with the non-preferred female before the 2^nd ^part of the tests (specific sperm competition risk treatments).

**Results:**

The strength of individual male preferences declined slightly also during the control treatment (1). However, this decrease was more than two-fold stronger in audience treatment (2), i.e., with non-specific sperm competition risk including the possibility for visual eavesdropping by the audience male. No audience effect was found in treatments (3) and (5), but a weak effect was also observed when the focal male had seen the previously preferred female sexually interact with a rival male (treatment 4; specific sperm competition risk).

**Conclusion:**

When comparing the two 'non-specific sperm competition risk' treatments, a very strong effect was found only when the audience male could actually observe the focal male during mate choice [treatment (2)]. This suggests that focal males indeed attempt to conceal their mating preferences so as to prevent surrounding males from copying their mate choice. When there is no potential for eavesdropping [treatment (3)], non-specific specific sperm competition risk seems to play a minor or no role. Our results also show that *P. mexicana *males tend to share their mating effort more equally among females when the resource value of their previously preferred mate decreases after mating with a rival male (perceived specific sperm competition risk), but this effect is comparatively weak.

## Background

An increasing body of literature considers the question of how mate choice is influenced by the social environment of the choosing individual (non-independent mate choice [1-7]). Traditionally, mate choice is viewed as an interaction and exchange of information between just two individuals, but recent studies have highlighted the role of social context for mating decisions [3,5,8-13] thus acknowledging that information can be public and may be used by individuals other than the intended receiver [14-18].

Animals use public information in various contexts [6,19-23]; for example, green swordtail (*Xiphophorus hellerii*) males are less likely to initiate a fight against observed winners [24,25]. Individuals may also use public information during mate choice to assess the quality of potential mates (e.g., [8,26-30]). For example, female crayfish (*Procambarus clarkii*) eavesdrop on male contests and prefer winners to losers [31]. Another widespread phenomenon is that individuals may copy the mate choice of others [8,29,32-35].

Audience effects occur when the presence of an observing (by-standing) individual leads to changes in the behavior of the observed individual(s) [36-43]. For example, swordtail (*Xiphophorus birchmanni*) males (Poeciliidae) court females more intensely in the presence of a male audience, suggesting that male courtship in that species has a dual function in mate attraction and to deter rivals [44], while male sticklebacks (*Gasterosteus aculeatus*) court females less in the portion of a test tank in which an audience male is visible [45].

Recent studies using a live-bearing fish (*Poecilia mexicana*) as a model found that the visual presence of a male competitor (i.e., an audience male) affects the strength of male mating preferences, with a weaker expression of preferences when the audience male observed the focal male [10,11]. How can this effect be explained? Theoretically, males could try to avoid aggressive interactions by moving away from the preferred female (see [10] for a discussion), which implies that aggressive behavior would play an important role in determining the expression of male mating preferences in the study species. This hypothesis received little support, because a very similar audience effect was found also in a population of *P. mexicana *with strongly reduced aggression (the cave molly [11]). Cave mollies naturally live in a dark and sulfidic habitat, and show very low body condition, so energy-limitation appears to have selected for reduced aggression ([11] for a discussion). A recent study [13] argued against another alternative interpretation, namely, the 'split-attention' hypothesis: if split-attention played a role, then also females should alter their mate choice decisions in the presence of a same-sex audience. Even though *Poecilia *females spent considerable time interacting with the audience female, no comparable decline in the expression of mating preferences was detected [13].

It was, therefore, argued that the adaptive significance of altered mate choice behavior in the presence of an audience is probably linked to an increased risk of sperm competition [42]. First, male competitors are likely to show the same intrinsic mating preferences (e.g., for large female body size: [10,11]; this study), so more equal allocation of mating efforts with respect to different female phenotypes may be a more profitable option under sperm competition risk. Hence, males should alter their mate choice under perceived sperm competition risk even if no rival observes them directly during mate choice. Secondly, a rival may observe the focal male and copy his choice at a later point in time [32,34], so males would benefit from concealing their interest in a particular female. (Male mate choice copying in poeciliids has probably evolved because sexual attention by a given male can be indicative of female receptivity [32]). In this scenario, males should alter their mate choice only when they are observed directly by an audience.

Here we provide a direct test whether males indeed adjust their mate choice behavior to the perceived risk of sperm competition. We gave focal males a choice between two different-sized females and scored association times near the two females as a measure of mating preferences (1^st ^part of the tests). The tests were repeated (2^nd ^part) under 'non-specific sperm competition risk', where an audience male was presented throughout the 2^nd ^part of the test (possibility for eavesdropping) or only before the 2^nd ^part of the tests (no possibility for eavesdropping), but could not interact with either female. We also designed treatments with 'specific sperm competition risk', where the focal male could observe one of the two females (either the initially preferred or the initially non-preferred one) sexually interact with a rival (see [46] for *Poecilia reticulata*; [47] for *Gambusia holbrooki*). We compared the decrease in strength of male preferences among the different treatments, which allowed us to disentangle the relative importance of (*a*) perceived specific and/or non-specific sperm competition risk and (*b*) mere sperm competition risk versus visual presence of an eavesdropping rival.

## Materials and methods

### Study organism and fish maintenance

Poeciliid fishes are livebearers and males use their transformed anal fin, the gonopodium, to transfer sperm. Females store sperm to fertilize several consecutive, monthly broods, and sperm competition is intense [48]. The Atlantic molly (*Poecilia mexicana*) is widespread in various streams, lakes and lagoons along the Central American Atlantic coast. Males typically form dominance hierarchies, where the dominant males (typically the largest) aggressively defend shoals of females [49]. While *P. mexicana *females have a cryptic coloration, large males show conspicuous black vertical bars and dominant males may even become completely black with yellowish to orange margins on the dorsal and anal fins. Smaller males are typically less conspicuous in coloration and attempt to sneak copulations in the absence of dominant males [49,50]. *Poecilia mexicana *males do not court females [51]; they almost constantly engage in either defending females from other males or attempting to mate [49].

The fish used in this study were descendants of animals collected in a coastal brackish lagoon near Tampico in central Mexico. Test fish came from large, randomly out-bred stocks. Two stocks were maintained at the Institute of Biochemistry & Biology of the University of Potsdam, and three at the Institute of Ecology, Evolution & Diversity in Frankfurt/M. Experiments were equally conducted at both universities. We reared mixed-sex stocks in aerated and filtered 100–200 l aquaria (comprising approximately 60–100 fish each) at 27–29°C. Artificial light was provided during a 12: 12 hrs light: dark cycle in addition to natural daylight entering the room through several windows. Aquaria were equipped with live and artificial plants and rocks. Fish were fed twice daily with commercial flake food and fish food tablets. We isolated focal males in 25 l tanks for 24 hrs prior to the tests to make sure that they were motivated to mate. We tested each focal male only once; however, due to the limited number of males available from our stocks, some males were also used as audience males after they had served as a focal male, but never on the same day. No male served as an audience more than once.

### Experimental design

#### General set-up

**Figure 1:**
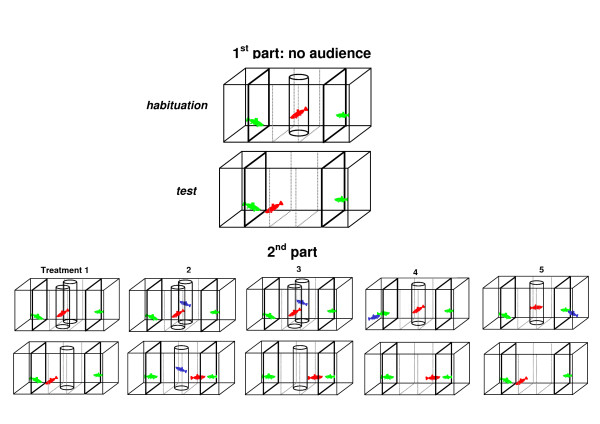
**Experimental set-up**. A focal male (*red*) was given a choice between two different-sized females (*green*). During the five different audience treatments, an audience/rival male (*blue*) was presented during or before the 2^nd ^part of the experiment. Each part consisted of two sequences of habituation and testing with switched side-assignments of the two stimulus females; for display purpose, only the first sequence of the 1^st ^and 2^nd ^part of a trial are depicted here. For details see main text.

The test tank (80 cm length × 30 cm width × 30 cm depth) was divided into five sections of equal size: two lateral compartments were divided by transparent Plexiglas partitions to hold the stimulus fish, the remainder was visually divided by marks drawn on the front into a central neutral zone and two lateral preference zones (Figure 1; [10]). All sides except the front wall were covered by black plastic foil. The tank was filled to 15 cm with aged tap water of 27–28°C and was illuminated by a 40 Watt incandescent lamp 35 cm above the tank in addition to the room illumination (two 100 Watt neon tubes on the ceiling of the experimental room). The water of the test tank was aerated between trials, but the air pump was turned off during the experiment. Prior to a test, two stimulus females (large: 46.0 ± 0.5 mm SL; small: 29.7 ± 0.4 mm SL) were haphazardly taken from a stock tank and introduced into one of the two stimulus compartments each. Each trial was conducted with another pair of stimulus females. Females may have been re-used in another trial, but it seems highly unlikely that the same stimulus pair combination (large and small) was used more than once. Then, we introduced a focal male (32.3 ± 0.4 mm SL) into a transparent Plexiglas cylinder (10 cm in diameter) in the center of the neutral zone and left the fish undisturbed for 5 minutes. After the habituation period, we gently lifted the cylinder by hand and initiated measurement of male association preferences. Trials were observed directly while the observer was sitting quietly approximately 2 m from the test tank. We measured the times the male spent in each of the two preference zones, i.e., near either female, during a 5-minute observation period. *Poecilia mexicana *males isolated for at least 24 hrs almost invariably attempt to mate [12], so association preferences were most likely sexual preferences, not just shoaling preferences. To account for potential side-biases, we placed the male into the cylinder again after the first observation period and interchanged the stimulus females. Measurement of male preferences was repeated after another 5 minutes of habituation. This episode consisting of two test units is henceforth called the 1^st ^part of a trial. We summed the times spent near both kinds of females during the two test units. Four trials were excluded based on our a priori definition of side biases (trials in which males spent more than 80% of their choice time in the same preference zone during the two test units [10]).

#### Audience treatments

Directly after the 1^st ^part of a trial, we repeated measurement of individual male preferences while an audience male (32.2 ± 0.4 mm SL) was presented during some of the five different audience treatments (see below; Figure 1). This allowed us to compare the strength of male preference before and after presentation of an audience, i.e., during the 1^st ^and 2^nd ^part of the tests (repeated measurements). Habituation, measurement of female association preferences and switching of side-assignments of the stimulus females between the two measurements was carried out as described above. Again, we summed up association times near either female during the two test units of the 2^nd ^part of a trial. The order of the five different treatments was random.

**Figure 2:**
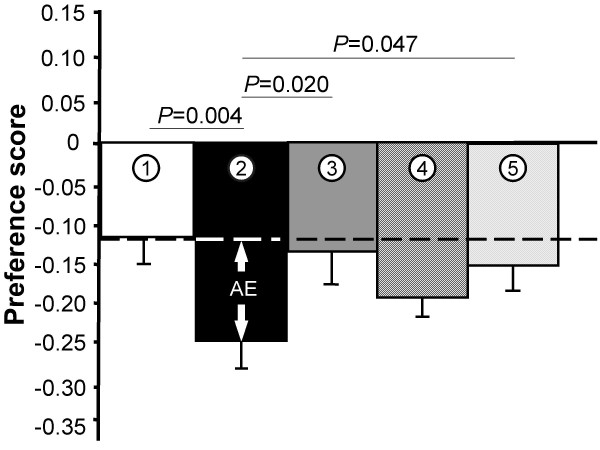
**Changes in the strength of male preferences during the five audience treatments [(1) no audience (control), (2) audience male was presented throughout 2^nd ^test period, (3) audience was presented only during habituation phase preceding the 2^nd ^test period, (4) like (3) but audience male could interact sexually with previously preferred female, (5) like (3) but audience male could interact sexually with previously non-preferred female]**. Shown are preference scores (percent time spent near initially preferred female during 2^nd ^part – time spent near the same female during 1^st ^part), such that negative values indicate that male preferences decreased during the 2^nd ^part of the tests. The difference between the control treatment (1) and treatment (2) can be interpreted as audience effect (*AE*). *P*-values refer to post hoc pair-wise Fisher's PLSD tests; only significant values are given.

To initiate this 2^nd ^part, we transferred the focal male back into the acclimation cylinder. During treatment (1), we presented an empty transparent Plexiglas cylinder without audience male in the central back of the neutral zone, equidistant to the two stimulus females. This 'baseline' treatment was conducted to test whether any changes in the expression of male preferences in the course of the experiment were truly due to the audience or whether the focal males' motivation to choose would generally decrease over time [13]. Only if the decrease of male preferences during the actual audience/sperm competition treatments (2–5) was stronger than in the baseline treatment (1) would this difference be interpretable as an audience effect (*AE *in Figure 2).

Treatment (2) was identical to our previous audience treatment [10,11,13]. We presented a conspecific audience male inside the transparent cylinder throughout the 2^nd ^part. The audience male was confined in its cylinder, so any direct physical interaction was ruled out.

In treatment (3) we created an experimental situation where the focal male also perceived non-specific sperm competition risk, but could not see the audience male during the actual mate choice trial. Hence, any effect of the focal male attempting to conceal his mating preferences so as to prevent the audience male from copying his mate choice could be ruled out. The audience male was presented inside the transparent Plexiglas cylinder during the two acclimation phases, but was gently removed before the focal male was released from his cylinder to exercise mate choice.

Finally, in treatments (4) and (5) we created an experimental situation where the focal male could observe the "audience" male (henceforth referred to as rival male) interact sexually with one of the two stimulus females (see [47] for a similar design using *Gambusia holbrooki*). The rival male was placed inside one of the female stimulus compartments during the first acclimation phase of the 2^nd ^part of the experiment. In half of the trials, the rival male could interact with the previously preferred female [treatment (4)], while in another half of trials it interacted with the previously non-preferred female [treatment (5)]. We scored sexual behaviors shown by the rival male (nipping at the female gonopore, a typical pre-mating behavior in mollies, and gonopodial thrusting) to make sure that the focal male would actually perceive sperm competition risk. All rival males exhibited sexual behavior [treatment (4), nipping: 43.8 ± 8.0, thrusting: 9.2 ± 2.0; treatment (5), nipping: 30.5 ± 7.8, thrusting: 6.3 ± 2.1].

### Statistical analysis

We scored a total of *N *= 145 trials [treatment (1), *N *= 30; treatment (2), *N *= 39; treatment (3), *N *= 27; treatment (4), *N *= 25; treatment (5), *N *= 24]. All statistical analyses were conducted using SPSS 12.0. Data are generally presented as means ± SE and were tested with Kolmogorov-Smirnov-tests for normality. All relative data were arcsine (square root)-transformed prior to statistical analysis.

To investigate the overall direction of male preferences, we first compared the times focal males spent near the large and small stimulus females using paired *t*-tests. Our major question was whether focal males would alter their individual choice decisions between the two parts of a trial, i.e., before and after presentation of an audience. We therefore compared the relative time males spent near the preferred female during the 1^st ^part of a trial [time spent in association with the preferred female/(time spent in association with preferred female + time spent in association with non-preferred female] with the relative time spent near the same (initially preferred) female during the 2^nd ^part of a trial. We compared relative association times before and after presentation of an audience (repeated measurement) in a repeated measures general linear model (rmGLM) using 'treatment' (five levels, see above) as independent variable. Due to decreasing motivation of the focal males to choose during the course of the two parts of the tests, slightly reduced expression of male preferences was predicted also for the control treatment (1) [13]. However, at least in treatment (2) a decline of male preferences beyond this baseline effect should be found (as reported in [10]). Based on previous studies on other poeciliid species [46,47], one would expect a strong decline in strength of male preferences also in the 'specific sperm-competition' treatment (4)–a male might actually switch to the previously non-preferred female after a rival had mated with his preferred mate. Altogether, this should result in a significant interaction effect of 'repeated measurement by treatment'.

For a *post hoc *comparison across treatments, we calculated a score as the difference between individual males' relative association times near the initially preferred female during the 2^nd ^part and relative association times near the same female during the 1^st ^part, such that no change in male preferences would lead to a score of zero, negative values would indicate that the focal males spent less time near the initially preferred female in the 2^nd ^part of a trial and positive values would indicate that males spent relatively more time near the initially preferred female. Scores were compared among treatments using pair-wise Fisher's protected least significant difference (PLSD) tests.

## Results

### Direction of male preferences

During the 1^st ^part of the tests, males showed a strong overall preference for the larger of the two stimulus females and spent on average 324 ± 12 s near the larger and 206 ± 11 s near the smaller female (paired *t*-test: *t*_144 _= 5.25, *P *< 0.0001). When testing for such a preference during the 2^nd ^part within each of the five different audience treatments, a statistically significant effect was seen only during the baseline treatment (1) (large: 313 ± 19 s, small: 221 ± 20 s; *t*_29 _= 2.85, *P *= 0.008), but not during treatments (2) (large: 224 ± 16 s, small: 195 ± 17 s; *t*_38 _= 1.05, *P *= 0.30), (3) (large: 253 ± 22 s, small: 261 ± 22 s; *t*_26 _= -0.21, *P *= 0.84), (4) (large: 263 ± 19 s, small: 261 ± 17 s; *t*_24 _= 0.05, *P *= 0.96), and (5) (large: 297 ± 20 s, small: 226 ± 19 s; *t*_23 _= 1.84, *P *= 0.079).

### Changes of individual male preferences

In the rmGLM comparing individual male preferences before and after presentation of the audience/rival male, a significant effect of the repeated measurement was detected (Table 1), indicating that the strength of male preferences declined from the 1^st ^to 2^nd ^part of the trials throughout treatments (Figure 2). However, a significant interaction effect of 'repeated measures by audience treatment' indicates that this decrease differed across audience treatments (Table 1).

**Table 1:** Results from rmGLM using the relative time spent near the preferred female during the 1^st ^part of the tests and relative time near the same (initially preferred) stimulus female during the 2^nd ^part as dependent variables (repeated measurement, *rm*).

	Effect	*df*	Mean square	*F*	*P*
Within-subjects effects	Rm	1	2.671	102.199	< 0.0001
	Rm × audience treatment	4	0.089	3.395	0.011
	Error	140	0.026		
Between-subjects effects	Audience treatment	4	0.147	2.888	0.025
	Error	140	0.051		

For audience treatments see main text and Figure 1.

We used a score expressing the decrease in the expression of male preferences to analyze this effect further. Qualitatively, the decrease was strongest during our "classical" audience treatment (2) [the decrease was 2.14-fold stronger than in the baseline treatment (1)]. The effect during treatment (4) was intermediate to treatments (1) and (2) [1.63-fold stronger than in the baseline treatment (1); Figure 2]. Pair-wise Fisher's PLSD tests confirmed that the decrease in male preferences during treatment (2) differed significantly from all other treatments with the exception of treatment (4) (*P *= 0.22; Figure 2). All other pair-wise comparisons yielded non-significant (*P *≥ 0.15).

## Discussion

In nature, communication is seldom binary like in classical, standardized mate choice experiments, but rather several individuals interact and communication networks prevail [5-7,52]. Our current study acknowledges the highly dynamic nature of poeciliid social aggregations (shoals) with often more or less stable female relationships, while males frequently move between shoals (guppy, *P. reticulata*: [53-56]). We simulated five different social contexts during which the focal males' mate choice was examined [(1) alone; (2) in the visual presence of another male; (3) in the presence of another male that could, however, not eavesdrop on the focal male's mate choice; and (4–5) after a rival male had copulated with one of the two females presented]. In our study, any decrease of male preferences stronger than in the control treatment (1) can be interpreted as an effect caused by the audience/rival male (*AE *in Figure 2).

Sperm competition in natural poeciliid populations can be intense [57,58]. Males respond to increased sperm competition (rearing at male-biased sex ratios), for example, by producing more sperm and mating more often [59,60]. Here, we investigated short-term, behavioral responses of *P. mexicana *males to sperm competition risk. We found weaker expression of mating preferences when an audience male could observe the focal male throughout the test [treatment (2)]. This effect could be due, primarily, to two reasons: neighboring males are likely to share intrinsic preferences for certain female phenotypes, such as large body size [61,62]. Mating preferentially with exactly those commonly preferred female types would intensify sperm competition once rivals are around. This simple form of the 'non-specific sperm competition' hypothesis, however, was not supported by our present data, because no audience effect was observed during treatment (3). On the other hand, also 'specific sperm competition' tended to affect the focal male's behavior. Focal males appear to have strategically adjusted their mating efforts by mating more equally with both females in treatment (4), where they saw the rival male interact sexually with the previously preferred mate. However, caution is required when interpreting this effect–the decrease in male preferences during this treatment was not significantly different from the control treatment (1).

So, why was the strongest effect seen in treatment (2)? We propose that males cease expressing mating preferences in the visual presence of a competitor to avoid being copied [32,34]. There is also some evidence that *P. mexicana *males may even attempt to deceive rivals about their mate choice [12]. Using an experimental design in which the focal male could interact freely with two different-sized females, it was shown that focal males directed their first sexual behavior almost exclusively toward the initially non-preferred female when an audience male was presented [12]. This was interpreted as an attempt by the focal male to actively mislead the audience male [12,42]. Again, this behavior appears to have evolved as a counter-adaptation in the face of male mate choice copying [42].

One might argue that any difference among treatments in the decrease of male preferences was driven solely by different times of presentation of the audience/rival males. Indeed, the audience male was presented throughout the entire 2^nd ^testing period in treatment (2), where the strongest effect was seen. It needs to be stressed though that the audience male was presented during both 5-minute acclimation phases of the 2^nd ^part in treatment (3)–still the effect was (at least qualitatively) weaker than in treatment (4), where the rival male was presented only during one (the first) 5-minute acclimation phase.

## Conclusion

Non-specific sperm competition risk appears to play a vital role when the audience male can actually eavesdrop on the focal male's mate choice. Hence, audience effects, as reported in previous studies [10,11] are best explained as an attempt by the focal male to conceal his interest in a particular female so as to prevent the audience male from copying his mate choice. By contrast, audience effects are probably not driven by a simple form of 'non-specific sperm competition risk' in a way that the presence of potential rivals that might later mate with the same female (treatment 3) would affect male mate choice. Also 'specific sperm competition' leads males to alter their mate choice, but this effect is comparatively weak. Our study in general highlights the important role of multidirectional visual communication events in poeciliid social networks for the expression of mating preferences.

## Competing interests

The authors declare that they have no competing interests.

## Authors' contributions

MZ, KM, CHS and CK carried out the behavioral tests. MP and MZ conducted the statistical analyses and wrote the first draft of the manuscript. RT, BS, MZ and MP were equally involved in implementing the project, and developing later drafts of the manuscript. All authors read and approved the final manuscript.
